# Inhibition of the β-carbonic anhydrase from the dandruff-producing fungus *Malassezia globosa* with monothiocarbamates

**DOI:** 10.1080/14756366.2017.1355307

**Published:** 2017-08-02

**Authors:** Alessio Nocentini, Daniela Vullo, Sonia Del Prete, Sameh M. Osman, Fatmah A.S. Alasmary, Zeid AlOthman, Clemente Capasso, Fabrizio Carta, Paola Gratteri, Claudiu T. Supuran

**Affiliations:** a Department Neurofarba – Pharmaceutical and Nutraceutical Section, University of Firenze, Firenze, Italy;; b Department Neurofarba – Pharmaceutical and Nutraceutical Section, Laboratory of Molecular Modeling Cheminformatics and QSAR, University of Firenze, Firenze, Italy;; c Polo Scientifico, Laboratorio di Chimica Bioinorganica, University of Firenze, Firenze, Italy;; d Istituto di Bioscienze e Biorisorse – CNR, Napoli, Italy;; e Department of Chemistry, College of Science, King Saud University, Riyadh, Saudi Arabia

**Keywords:** Carbonic anhydrase, β-CA-class enzyme, monothiocarbamate, inhibitor, *Malassezia globosa*

## Abstract

A series of monothiocarbamates (MTCs) was investigated for the inhibition of the β-class carbonic anhydrase (CAs, EC 4.2.1.1) from the fungal parasite *Malassezia globosa*, MgCA. These MTCs incorporate various scaffolds, among which aliphatic amine with 1–4 carbons atom in their molecule, morpholine, piperazine, as well as phenethylamine and benzylamine derivatives. All the reported MTCs displayed a better efficacy in inhibiting MgCA compared to the clinically used sulphonamide drug acetazolamide (K_I_ of 74 μM), with K_I_s spanning between 1.85 and 18.9 μM. The homology model of the enzyme previously reported by us was used to rationalize the results by docking some of these MTCs within the fungal CA active site. This study might be useful to enrich the knowledge of the MgCA inhibition profile, eliciting novel ideas pertaining the design of modulators with potential efficacy in combatting dandruff or other fungal infections.

## Introduction

Carbonic anhydrases (CAs, EC 4.2.1.1) are metalloenzymes present in all life kingdoms which catalyse the hydration of carbon dioxide to bicarbonate, with seven genetically distinct families described to date in various organisms, the α-, β-, γ-, δ-, ζ-, η-and θ-CAs^1–13^. The inhibition of many such enzymes, present in mammals (in which there are 16 different isoforms)^14–24^ or in various pathogens (fungi^10^^,^^25–32^, bacteria^2^^,^^4^^,^^33–37^, or protozoa)^6^^,^^8^^,^^10^^,^^38^^,^^39^ may be exploited pharmacologically^1^^,^^4^^,^^10^^,^^16^^,^^40^. Sulphonamides are the main class of CA inhibitors (CAIs)^1^^,^^14^^,^^41–46^, but they show many side effects, and for this reason many efforts were made in the last decade in order to develop alternative classes of inhibitors^14^^,^^15^^,^^47^. In particular, the inorganic anions^5^^,^^13^^,^^28^, phenols^48^, polyamines^49^, and dithiocarbamates/xanthates/monothiocarbamates (MTCs)^50–55^ represent interesting cases, which have been investigated in detail by kinetic and crystallographic studies. Such studies allowed a detailed understanding of the inhibition mechanisms with these classes of compounds and led to interesting drug design campaigns as well as the discovery of CAIs with a good selectivity ratio for inhibiting enzyme classes or isoforms of pharmaceutical interest^1^^,^^4^^,^^14^^,^^17^.

Recently, we cloned and characterized a β-CA in the pathogenic fungus provoking dandruff *Malassezia globosa*, MgCA^26–28^. This enzyme was shown to be an effective catalyst for the physiologic reaction, CO_2_ hydration to bicarbonate and protons, whereas its inhibition with sulphonamides led to growth defects of the fungus *in vivo*. Such results showed for the first time relevant antidandruff effects by targeting MgCA, which were equivalent to those of the standard azole drug ketoconazole^26^. Furthermore, we have subsequently developed a different cloning and purification strategy for this enzyme^27^, and showed that apart sulphonamides, anions, dithiocarbamates and amino acids also constitute interesting modulators of its activity^27^^,^^28^. Finding non-sulphonamide, effective CAIs targeting MgCA seems to be a challenge not very easy to address, and this is the reason why we decided to investigate also the MTCs for this scope, as this class of CAIs was only recently reported for its interactions with human (h), α-class enzymes^54^. Indeed, *M. globosa*, one of the main fungi belonging to this genus which infects humans^56–61^, is difficult to eradicate and has developed significant resistance to most azole antifungals^62^.

## Materials and methods

### Chemistry

Compounds **1–17** used in the experiments were reported earlier^54^.

### CA assay

An applied photophysics stopped-flow instrument has been used for assaying the CA catalysed CO_2_ hydration activity^63^. Bromothymol blue (at a concentration of 0.2 mM) has been used as indicator, working at the absorbance maximum of 557 nm, with 10–20 mM TRIS (pH 8.3) as buffer, and 20 mM NaBF_4_ for maintaining constant the ionic strength, following the initial rates of the CA-catalysed CO_2_ hydration reaction for a period of 10–100 s. The CO_2_ concentrations ranged from 1.7 to 17 mM for the determination of the kinetic parameters and inhibition constants. For each inhibitor at least six traces of the initial 5–10% of the reaction have been used for determining the initial velocity. The uncatalysed rates were determined in the same manner and subtracted from the total observed rates. Stock solutions of inhibitor (10 mM) were prepared in distilled-deionized water and dilutions up to 0.01 µM were done thereafter with the assay buffer. Inhibitor and enzyme solutions were preincubated together for 15 min at room temperature prior to assay, in order to allow for the formation of the E–I complex. The inhibition constants were obtained by non-linear least-squares methods using the Cheng–Prusoff equation whereas the kinetic parameters for the uninhibited enzymes from Lineweaver–Burk plots, as reported earlier^64–71^, and represent the mean from at least three different determinations. MgCA was a recombinant protein, obtained and purified by a diverse procedure as the one reported earlier^27^.

### Molecular modelling studies

The dimeric form of the homology built model of MgCA^55^ was prepared for docking using the Schrodinger preparation wizard protocol that consists in preliminary pretreatment by adjusting the bond orders, metal ions and cofactors, evaluating the ionization states, adding hydrogen atoms, refining loop region and energy minimization^72^.

3D ligand structures were prepared by Maestro^73^, evaluated for their ionization states at pH 7.4 ± 1.0 with Epik^74^. The atomic electrostatic charges of the ligands were computed with Jaguar^75^ fitting them to an electrostatic potential calculated at the B3LYP/6–31 G*+ level of theory. ESP atomic charges were used in docking simulations.

Grids for docking analysis were centered in the centroid of the catalytic cavity residues. Docking studies were carried out with the program Glide^76^. Grids for docking were centered in the centroid of the complexed ligand. The standard precision (SP) mode of the GlideScore function was applied to evaluate the predicted binding poses. The pictures were generated with Maestro^73^.

## Results and discussion

### Chemistry

The rationale of this work was to investigate whether MTCs, ion analogue to the DTCs previously investigated^55^, show effective inhibitory action against MgCA, a β-class CA. It should be stressed that the MTCs were only recently reported as a new class of CAIs by this group^54^. Similarly to DTCs and to trithiocarbonate, they possess a zinc-binding group (ZBG) which may coordinate effectively to the catalytically crucial metal ion from the MgCA active site, which is a Zn(II) ion^26–28^. The ZBG is of the CS_2_^–^ type for trithiocarbonate and DTCs^50–55^, and of the COS^–^ type for MTCs^54^. X-ray crystallography was successful so far only for adducts of the human (h) isoform hCA II with some DTCs^51^, which clearly demonstrated that one sulphur of the ZBG is coordinated monodentately to the Zn(II) ion whereas the scaffold of the inhibitor participates in many favourable interactions with the enzyme active site. For MTCs, computational studies showed a similar behavior^55^, with the COS^–^ ZBG coordinating (through the sulphur atom) to the metal ion from the enzyme active site. Recently, we also developed a homology model for MgCA alone and in complex with DTCs^55^, which showed that also for the β-class enzyme, DTCs bind in a similar manner as for α-CAs, with the sulphur atom of the ZBG directly bound to the Zn(II) ion. Here, we extend the previous studies to MTCs in order to understand whether the COS^–^ ZBG may also lead to effective MgCA inhibitors. The series of compounds **1–17** previously reported^54^, has been used for the enzyme inhibition measurements and includes MTCs **1–15**, the trithiocarbonate **16** and the xanthate **17** (Table 1). These compounds incorporate aliphatic, aromatic and heterocyclic scaffolds, in order to explore the structure-activity relationship (SAR) of a varied chemical space for inhibition of MgCA with this class of compounds.

**Table 1.** hCA I, II and MgCA inhibition data with MTCs **1−15**, trithiocarbonate **16** and xanthate **17** by a stopped-flow CO_2_ hydrase assay^63^.


			K_I_^a^		
No.	R	R_1_	hCA I (nM)	hCA II(nM)	MgCA (µM)
**1**^b^	H		891	26.7	14.1
**2**^b^	H		>2000	43.7	18.9
**3**^b^	H		>2000	35.0	7.81
**4**^b^	*n*-Pr	*n*-Pr	>2000	46.7	1.85
**5**^b^	*n*-Bu	*n*-Bu	909	>2000	7.52
**6**^b^	*i*-Bu	*i*-Bu	681	43.0	8.61
**7**^b^	Et	*n*-Bu	700	>2000	5.26
**8**^b^	Me		827	44.5	9.16
**9**^b^	Me	Bn	>2000	>2000	7.61
**10**^b^	–(CH_2_CH_2_)–O–(CH_2_CH_2_)–		569	>2000	7.65
**11**^b^	–(CH_2_CH_2_)–NH–(CH_2_CH_2_)–		876	22.4	7.41
**12**^b^	–(CH_2_CH_2_)–N(4–F–C_6_H_4_)–(CH_2_CH_2_)–		895	46.8	8.33
**13**^b^	–(CH_2_CH_2_)–N(4–CF_3_–C_6_H_4_)–(CH_2_CH_2_)–		>2000	43.6	4.22
**14**^b^	–(CH_2_CH_2_)–N(3–Cl–C_6_H_4_)–(CH_2_CH_2_)–		686	>2000	15.9
**15**^b^	–(CH_2_CH_2_)–N(CH_2_CONHC_6_H_11_)–(CH_2_CH_2_)–		949	45.9	6.13
**16**^b^	Bn	–	4.1	0.70	8.12
**17**^b^		–	64.1	5.4	16.2
AAZ			250	12	74

a Mean from three different assays, by a stopped flow technique (errors were in the range of ±5–10% of the reported values); ^b^hCA I and hCA II data from Ref.^14f^.

### CA inhibition

Inhibition data with compounds **1–17** against the hCA isoforms (off targets) hCA I and II, as well as MgCA, with the sulphonamide inhibitor acetazolamide (**AAZ**) as standard^63^, are shown in Table 1.

The following SAR for the inhibition of MgCA with compounds **1–17** can be compiled from the data of Table 1:

(i) Independently from the nature of the substituents attached to the MTC nitrogen, all reported derivatives showed better MgCA inhibitory properties than the clinically used sulphonamide drug **AAZ** (K_I_ of 74 μM), with K_I_s ranging between 1.85 and 18.9 μM.

(ii) A rather flat SAR can be extrapolated from the data reported in Table 1. Indeed, the nature of the *R* and *R*_1_ groups demonstrated to possess a limited influence on the inhibitory effectiveness of compounds **1–17**, which allegedly mainly lean on the ZBG interactions with the Zn ion and the residues nearby. On the other hand, it is necessary to stress the importance of lipophilic moieties (*R* and *R*_1_) present in the scaffold of the inhibitors, which may favour the overall interactions within the catalytic cleft, allowing by far a better inhibitory efficacy compared to simple inorganic anions (such as trithiocarbonate, CS_3_^2–^ which was the real lead for designing DTCs and MTCs as CAIs)^54^.

(iii) The di-*n*-propyl derivative **4** demonstrated the best efficacy in inhibiting the enzyme among the reported MTCs (K_I_ of 1.85 μM), sign that two short, unbranched aliphatic chains could better fit within the lipophilic pockets of the active site. The two secondary MTCs **1** and **2** were observed to generally possess slightly worse inhibitory potency (K_I_ of 14.1 and 18.9 μM) compared to **4** and the remaining tertiary MTCs, showing comparable K_I_s (ranging between 4.22 and 9.16 μM). The only exception was represented by derivative **14**, which incorporate a N-(3-Cl-phenyl)-piperazine moiety, this compound being in fact the less active tertiary MTC investigated here. In detail, the presence of a Cl atom in the *meta* towards the heterocyclic ring could elicit a steric hindrance within the MgCA binding pocket that reduced the K_I_ to 15.9 μM. Finally, whereas the benzyl trithiocarbonate **16** possessed a K_I_ comparable to those of most MTCs (K_I_ of 8.12 μM), the phenethyl xanthate **17** showed a roughly two-fold reduced efficacy (K_I_ of 16.2 μM).

(iv) The effectiveness of MTCs in inhibiting the MgCA was shown to be scarcely reduced, but anyhow comparable, to that of the DTCs investigated earlier by us^55^. Diversely, the inhibition profile of the MTCs against MgCA was very poor in comparison to that of the human isoforms, which were more sensitive to this class of inhibitors. Notwithstanding no MgCA-selective inhibitors were found, the MTCs have been validated as a new class of MgCA inhibitors, and it is worth that they should be considered, alongside with the DTCs, as attractive for further investigations to discover more potent and selective fungal CAIs.

### Molecular modelling studies

**Figure 1.**
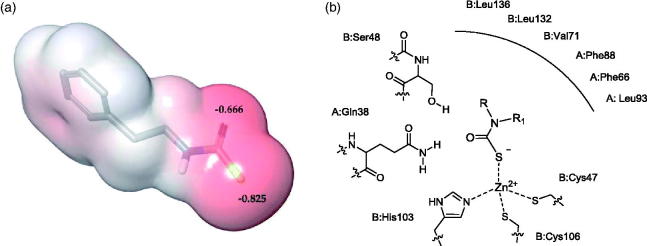
(a) ESP atomic charges of **2** derived from a B3LYP/6–31 G*^+^. Red colour represents negative values of the electrostatic potential (b) Schematic representation of the binding mode of MTCs into the MgCA active site.

Docking simulations were performed to elucidate the binding mode of MTCs within the MgCA active site. Four inhibitors from Table 1 (compounds **2, 8**, **9** and **10**) endowed with acceptable inhibitory properties and a varied structure were selected as representatives of the synthesized MTCs. These derivatives were submitted to quantum mechanics optimization (B3LYP/6–31 G*^+^) in order to compute the charge distribution and optimal geometry, prior to dock the molecules into the recently developed homology-built model of MgCA^55^. According to previously reported evidence^54^^,^^77^, points of high electron density surface are located close to the sulphur atom of the MTC (as in the DTCs previously investigated). The active site of the enzyme comprises residues from the two monomers (chain A and B) that form the quaternary structure, and the catalytic zinc ion is coordinated by the side chains of C47, H103 and C106. The lowest energy docking solutions suggest that the fourth Zn coordination position can be occupied either by sulphur or oxygen atoms of the MTC inhibitor. However, based on the findings obtained by QM calculation (Figure 1(a)) and on the previous spectroscopic and crystallographic studies^77^, which agreed in indicating that the negative charge distribution is mainly localized on sulphur, poses were selected in which the sulphur atom binds in tetrahedral coordination geometry to the catalytic zinc ion from the enzyme active site. The oxygen atom of the MTC moiety was, on the other hand, found in H-bond distance from residues S48 (chain B) and Q38 (chain A), depending on the selected pose (Figure 1(b)).

**Figure 2.**
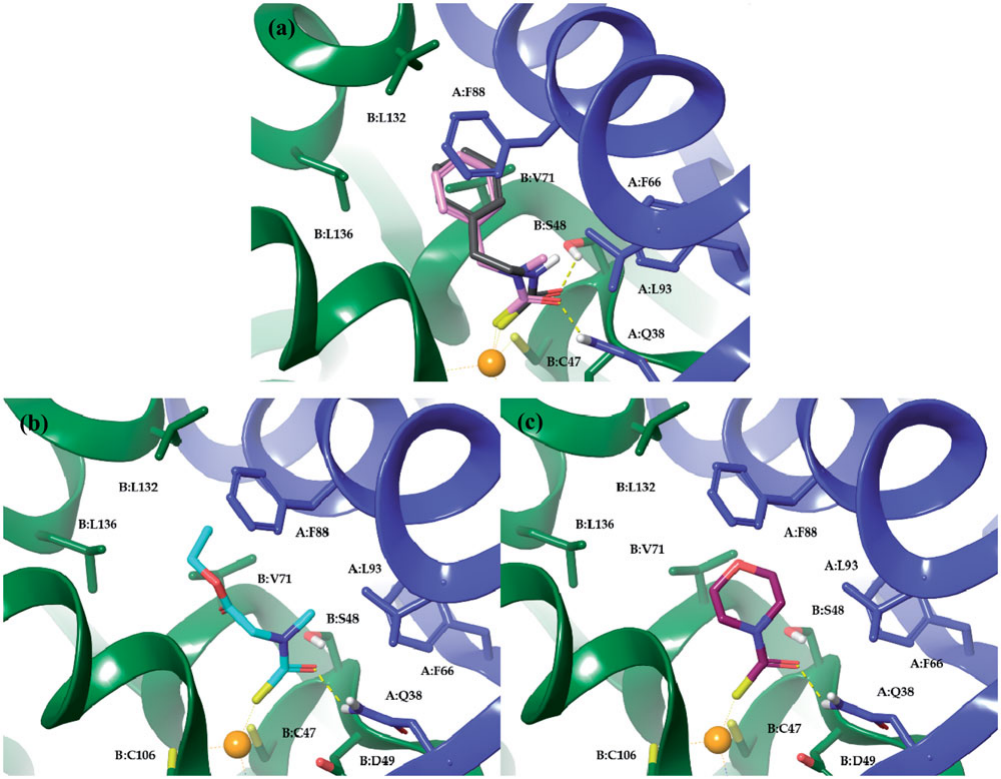
Docked orientations of compounds **2** and **9** (a); **8** (b) and **10** (c) within MgCA active site. Monomer A and B are coloured blue and green, respectively.

The scaffold fragments of the four derivatives accommodate into a hydrophobic pocket defined by residues from both monomers. π–π interactions occur between the phenyl moieties of derivatives **2a** and **9a** and the side chain of F88(A). The benzyl and phenethyl tails of these derivatives were further stabilized by the π-alkyl interactions established with the aliphatic side chain of V71(B) and L132(B) (Figure 2(a)). These same three residues and L136(B) were involved in hydrophobic interactions with the ethyl group of the ester function of **8** (Figure 2(b)). CH···π interactions were also observed for the *N*-methyl group of the zinc-binding group moiety of **8** and **9** and the side chains of F66 and L93 from monomer A. Alkyl- and π-alkyl interactions were also observed for the morpholine ring of **10** and the side chains of V71(B) and F88(A), respectively (Figure 2(c)).

Compared to the predicted binding mode of DTCs, which form H-bond with both S48 (chain B) and Q38 (chain A) residues, the oxygen atom of the MTC was able to bind only to the side chain OG atom of S48 or NE2 atom of Q38. Hence, it is reasonable to hypothesize that the shorter length of the CO bond (1.25 Å) compared to that of the CS (1.75 Å) one^77^ may contribute to the generally worse inhibitory profile of MTCs compared to DTCs.

## Conclusions

Kinetic and computational approaches were applied to investigate a series of MTCs as novel inhibitors of the β-class carbonic anhydrase from the fungal parasite *M. globosa*, a validated anti-dandruff drug target^55^^,^^78^. All the reported MTCs displayed better MgCA inhibition profile than to the clinically used sulphonamide drug acetazolamide (K_I_ of 74 μM), with K_I_s spanning between 1.85 and 18.9 μM. Docking procedures were applied to the homology model of the enzyme we previously reported to shed light on the binding mode the MTCs exhibited within the fungal CA active site. This study might be of help to better decipher the MgCA inhibition profile, raising the discovery of novel modulators with potential efficacy in combatting dandruff or other fungal infections.
